# Plasma-assisted synthesis and high-resolution characterization of anisotropic elemental and bimetallic core–shell magnetic nanoparticles

**DOI:** 10.3762/bjnano.5.54

**Published:** 2014-04-14

**Authors:** M Hennes, A Lotnyk, S G Mayr

**Affiliations:** 1 Leibniz-Institut für Oberflächenmodifizierung e.V., Permoserstr. 15, 04318, Leipzig, Germany; 2 Translationszentrum für Regenerative Medizin, Universität Leipzig, 04103 Leipzig, Germany; 3 Fakultät für Physik und Geowissenschaften, Universität Leipzig, 04103 Leipzig, Germany

**Keywords:** bimetallic magnetic nanoparticle, core–shell, magnetron sputtering, plasma gas condensation

## Abstract

Magnetically anisotropic as well as magnetic core–shell nanoparticles (CS-NPs) with controllable properties are highly desirable in a broad range of applications. With this background, a setup for the synthesis of heterostructured magnetic core–shell nanoparticles, which relies on (optionally pulsed) DC plasma gas condensation has been developed. We demonstrate the synthesis of elemental nickel nanoparticles with highly tunable sizes and shapes and Ni@Cu CS-NPs with an average shell thickness of 10 nm as determined with scanning electron microscopy, high-resolution transmission electron microscopy and energy-dispersive X-ray spectroscopy measurements. An analytical model that relies on classical kinetic gas theory is used to describe the deposition of Cu shell atoms on top of existing Ni cores. Its predictive power and possible implications for the growth of heterostructured NP in gas condensation processes are discussed.

## Introduction

Due to their size, novel physical properties and the possibility of contactless manipulation, magnetic nanoparticles can be employed as powerful nanotools in many areas of biology, biophysics and medicine [1]. Possible applications include their use as contrast agents for cell tracking via magnetic resonance imaging (MRI) [2], as colloidal mediators in cancer therapy (hyperthermia) [3] or as nanocarriers for targeted drug delivery [4]. Unfortunately, many ferromagnetic materials are prone to strong oxidation (thereby losing their magnetic properties over time) and turn out to be highly cytotoxic, a knock-out criterion for any application in life sciences. The synthesis of well designed nanoalloys [5], combining two and more metals at the nanoscale, might circumvent this problem. Core–shell nanoparticles (CS-NPs) that are composed of an inert metallic layer of several nanometer thickness covering a magnetic core, can prohibit the latter from oxidation and prevent the release of toxic ions in solution. While a plethora of wet-chemical methods has been designed for the synthesis of heterostructured magnetic particles [6], less is known about possible issues of methods that are based on inert-gas condensation, in which nanoparticles grow out of a supersaturated metal vapor. Yet, gas phase techniques possess several advantages over their chemical counterparts: the high purity of the resulting samples, high throughput in continuous operating mode, and easy integration into other UHV manufacturing/analysis steps. Although early experiments used inert gas condensation in combination with thermal evaporation [7], magnetron sputtering at high pressures soon emerged as a superior technique for the production of large amounts of nanoparticles with very narrow size distribution, a unique advantage for further fundamental and applied research [8–9]. Nevertheless, relatively few studies dealing with the precise tailoring of heterostructured particles in plasma gas condensation setups have been performed yet [10–12], and many publications remain mostly focused on the tuning of size and shape of selected elemental particles [13–19].

When concentrating on the synthesis of CS-NPs in the gas phase, two general approaches are practicable. On the one hand, self organizational properties of matter at the nanoscale can be exploited. Immiscibility of the involved components combined with pronounced differences in surface energies can result in CS structures through a single step process relying on the creation of a bimetallic metal vapor [11,20]. Nevertheless, it is doubtful that this approach will be employable for arbitrary binary alloys with miscibility gap. Indeed, extensive computational studies reveal that equilibrium segregation patterns of immiscible metallic components only seldom result in rotationally symmetric CS-structures [21–23]. Immiscibility was even predicted to be entirely suppressed in NPs [24–25]. The second approach, a two step synthesis process in which the core is produced first and subsequently covered by the vapor atoms of a second metal species therefore seems a promising alternative.

For systematic experimental studies of these scenarios, Cu/Ni constitutes an interesting model system with a moderate miscibility gap in the bulk solid phase (*T*_c_ = 630 K) [26] and a moderate difference in the surface energies between Cu and Ni (*γ*_Cu_ = 1.78 J/m^2^ and *γ*_Ni_ = 2.37 J/m^2^) [27]. Recent studies have put Cu/Ni nanoparticular phase diagrams under scrutiny: Thermodynamic equilibrium configurations of nanoclusters were predicted to depend on concentration and structure, and no indications for rotationally symmetric CS configurations were found [22–23]. In addition, the properties of the Cu/Ni system compare reasonably well with the Au/Ni system, which –when in the shape of magnetic core/inert shell nanoclusters– is highly promising as easily functionalized carrier in biomedical environments. Although our studies, at the present stage, are targeted at a fundamental physical understanding, they also can be regarded as a testbed to link to highly attractive future applications. Unfortunately, experimental studies on Cu/Ni or other bimetallic particles with weak miscibility gap are not available to date.

Here, without loss of generality, we therefore explore the capacity of a plasma gas condensation setup to produce out-of-equilibrium CS structures. After presenting experimental details in the first section, we will focus on several key aspects of gas phase synthesis of nanoparticles in the second part. The third and fourth part of this paper are devoted to the presentation and discussion of our results. The last section will consist of a summary and a conclusion.

## Experimental

### UHV setup and operating conditions

**Figure 1:**
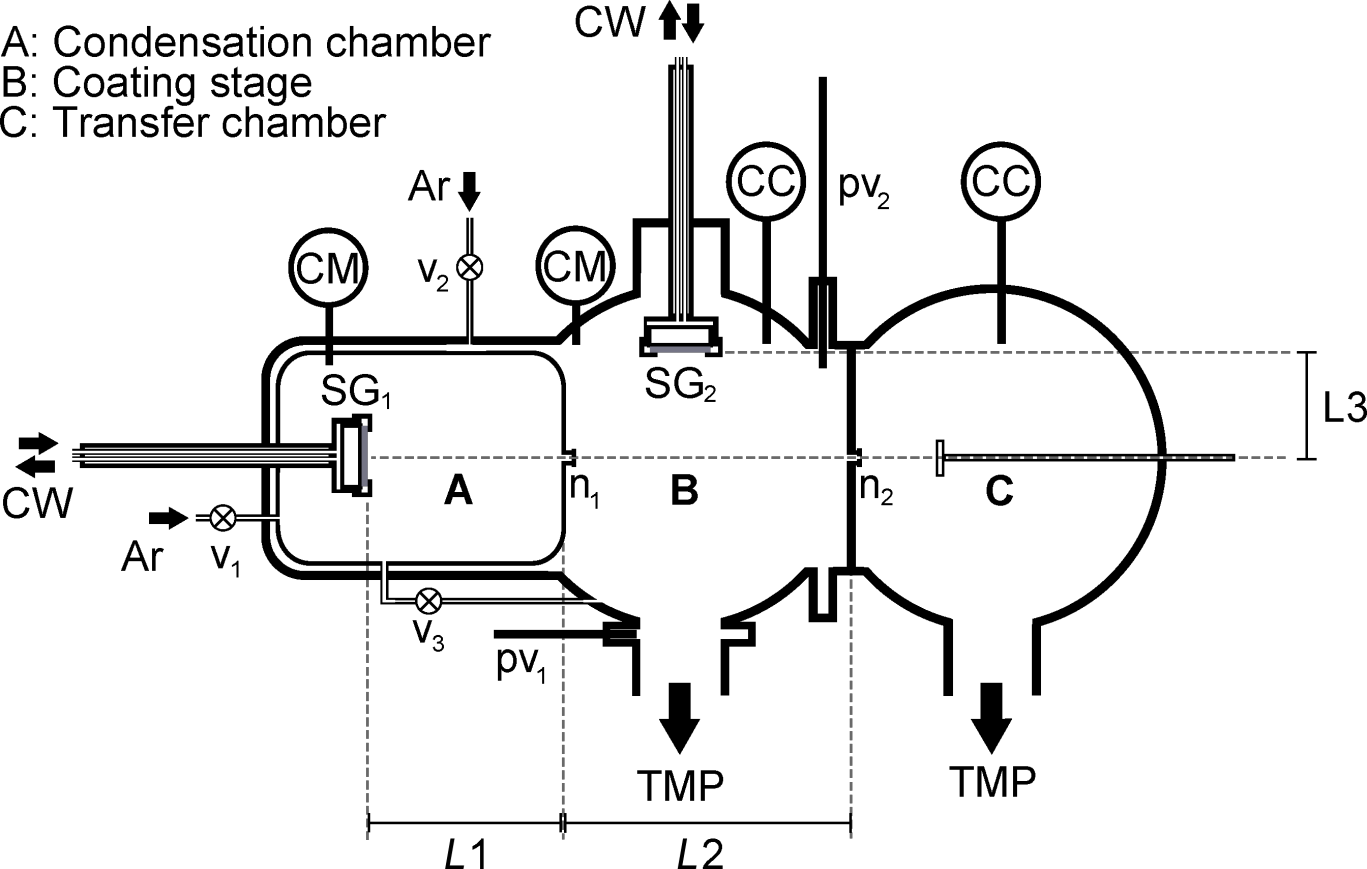
Schematic drawing of the home-built UHV setup used in the present study. CM: capacitance manometers, CC: cold cathode, TMP: turbo molecular pump, CW: cooling water, v: valve, pv: gate valve, SG: sputter gun, n: nozzle.

A sketch of the UHV setup used in the present study can be found in Figure 1. The apparatus is divided into three main regions: a condensation vessel (A), a coating stage (B) and a transfer chamber (C), connected by two nozzles n_1_ and n_2_. Argon 5.0 (99.999%) is used as inert gas and additionally cleaned with an O_2_ purifier (Air Liquide) so that the final oxygen content in the stream falls below 0.1 ppm, the partial pressure of contaminants therefore being roughly the same order of magnitude as the initial concentration in the evacuated vessels. Ar flow into A is controlled manually by a leak valve v_1_ (Balzers 135) while an additional valve v_2_ (MKS 248) regulates the stream into vessel B. The pressure in these two chambers can therefore be adjusted separately. The gas transport is maintained by differential pumping using two turbo molecular pumps (Varian TV 301) with a pumping capacity of 300 L/s. Two gate valves are employed to switch between gas flow configurations that enable pumping towards base pressures *p* ≈ 10^−7^ mbar (v_3_ and pv_1_ open, pv_2_ closed) and particle transport to C (v_3_ and pv_1_ closed, pv_2_ open). In chamber C the substrate is mounted on a rotating sample holder allowing for multiple deposition within one run without venting the chamber. The pressure in the chambers is monitored with the help of cold cathodes at pressures below 10^−3^ mbar (Pfeiffer IKR 251) and capacitance manometers (MKS Baratron 122A and 220BHS) when working in the mbar regime. Metal vapors are generated with the help of two 2-inch DC magnetron sputtering guns (AJA A-320) that can be operated up to pressures as high as 2 mbar. Both magnetron guns have additionally been equipped with a welded bellow to allow for a displacement of the sputter gun under UHV conditions. All particles generated with the present setup were analyzed after transport through air. Scanning electron microscopy (SEM) studies were performed with a Zeiss Ultra 55 (acceleration voltage *U* = 15 kV). High resolution transmission electron microscopy (HRTEM) investigation was done using a probe-Cs corrected FEI Titan3 G2 60-300 microscope operating at 300 kV acceleration voltage. Energy dispersive X-ray (EDX) analysis was performed by using a FEI SuperX detector with high visibility low-background FEI holder. The data was collected and evaluated with Bruker software. For EDX measurements, the microscope was set to STEM mode. The beam current was 200 pA. The nanoparticles were directly deposited on Cu grids covered by lacey carbon.

### Particle synthesis

Sputtering of the target material in chamber A leads to the ejection of metal atoms with typical mean energies in the eV range for the setup presented here. Subsequent thermalization in the buffer gas is fast and has been predicted to occur after few collisions, as shown analytically and with the help of Monte-Carlo (MC) simulations [28]. The cooling of the metal vapor is dependent on the background gas pressure. In our experiment, atoms are expected to be thermalized after less than a millimeter in chamber A, and less than a centimeter in chamber B, thus in a region close to the target with regard to chamber dimensions. When the metal vapor reaches sufficient supersaturation, the growth of small nuclei occurs. Following classical nucleation theory, these nuclei have to overcome a critical size to become thermodynamically stable and then further grow while moving through the condensation chamber towards the nozzle. The NPs can additionally get covered by a supplementary coating layer in chamber B, while traveling through the metallic vapor generated by SG_2_, which is carefully kept below supersaturation. Finally, particles are extracted in C, where they are deposited onto a Si substrate or TEM grids for further analysis.

### Gas dynamics and NP transport

Particle synthesis and growth is inherently connected to gas dynamics in the chamber. Indeed, as described above, the Ar background gas serves a threefold purpose: it is used as ionizable species for sputtering, as thermal reservoir for generation of the supersaturated metal vapor and finally, for convective particle transport. Working in the millibar-range allows for the application of standard continuum gas dynamics equations, as the mean free path of the gas is found well below chamber dimensions. With pressure differences between subsequent chambers exceeding the critical ratio of 0.487, the flow will be choked, which facilitates the calculation of Ar mass flow by using [29]

[1]
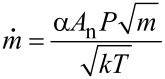


with *k* being the Boltzmann constant, *T* the temperature, *m* the mass of the Ar atoms, *A*_n_ the cross sectional area of the nozzle, *P* the upstream pressure and *α*, a numerical coefficient derived from


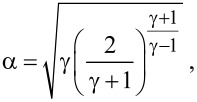


where *γ* is the ratio of heat capacities and equals 5/3 for the gas used herein. For our experimental setup, the total mass flow was calculated to be equal to 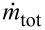 = 1.3·10^−5^ mol/s (*r*_n2_ = 1.25 mm, *P*_A_ = 1.51 mbar, *P*_B_ = 0.35 mbar, *T* = 300 K). The use of mass conservation permits the calculation of the convective velocity of the gas [13]

[2]
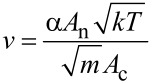


with *A_c_* being the cross sectional vessel area (*A*_c_ = 3.1·10^−2^ *m*^2^). For our setup, resulting approximate travel velocities between 1.5 and 3.0 cm/s for chamber A and B, respectively, have been calculated.

Although playing a major role in sputtering, the influence of the plasma is usually neglected in the analysis of particle growth and transport as the degree of ionization is expected to be low in the operating regime described in the present paper. In contrast, an eventual heating of the gas through thermalization of the metal vapor has to be considered [16,19]. A simple thermodynamic analysis of energy fluxes in the chamber can be used to find out whether Ar heating will be prominent or not. Assuming steady state conditions to be reached and neglecting energy loss at the chamber walls, the following identity can be derived

**Figure 2:**
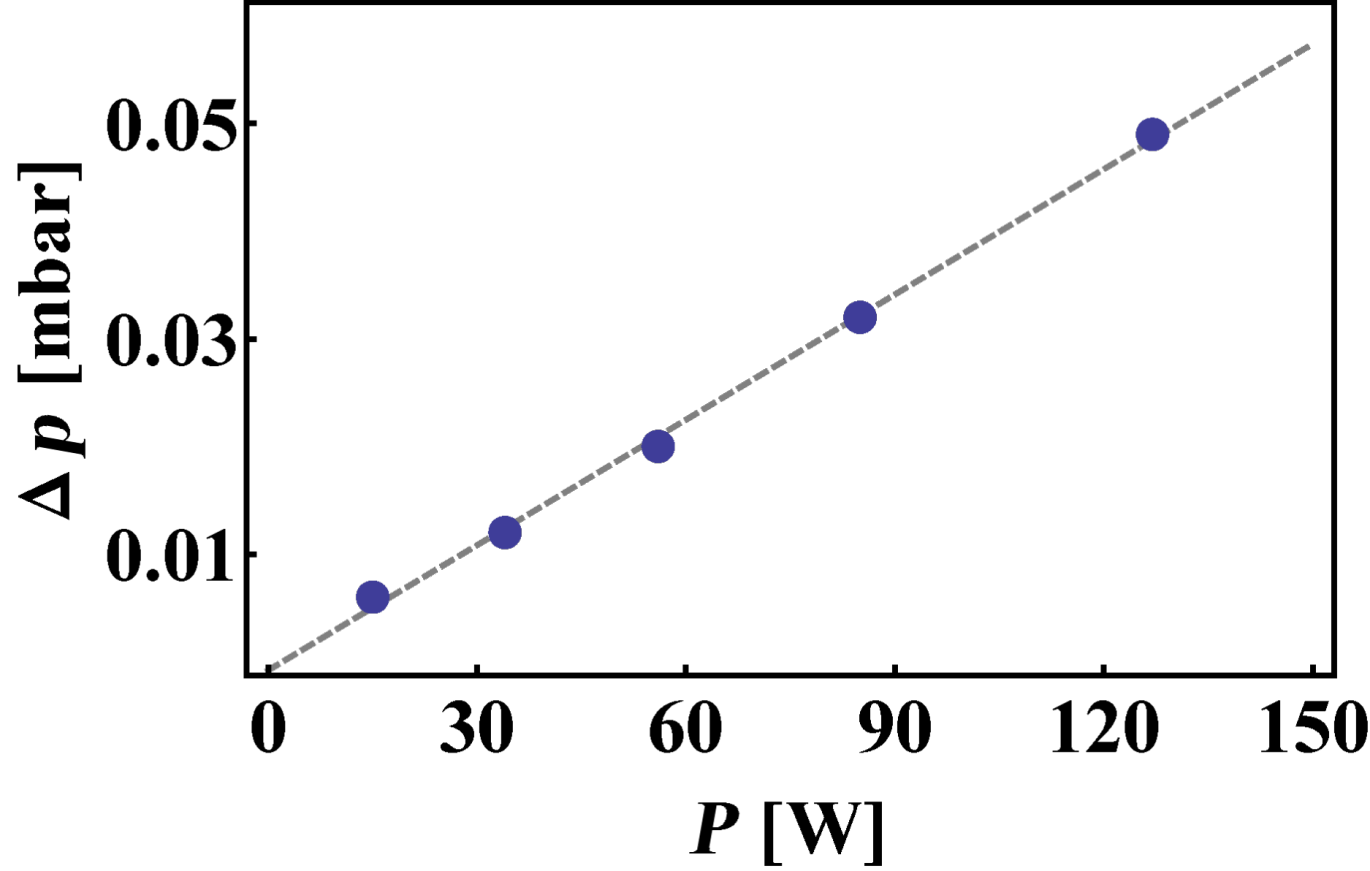
Measured linear pressure increase in chamber A as a function of magnetron power.

[3]
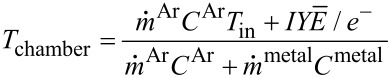


with *I* being the electrical current and *Y* the sputter yield, 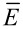 the average energy of the sputtered metal atoms and *C* the heat capacity of a monoatomic gas. Interestingly, the calculation of the above temperature yields erroneously high values contrasting with the measured pressure increase in chamber A observed when switching on the magnetron gun (Figure 2).

This quantity hardly exceeds 5%, even at high powers and might be considered a measure for overall gas heating in vessel A. This suggests that a) full thermalization of the sputtered species, especially of high energy metal atoms is not achieved and/or b) one is eventually overestimating the sputter yield by assuming that Ar ions get fully accelerated by the applied voltage. Indeed, energy loss mechanisms, such as elastic collisions and charge transfer during sputtering, might play a crucial role in our setup, in which the mean free path of gas atoms can drop well below the extension of the cathode dark space. This will again play a role in the discussion part of the present work.

## Results

### Elemental Ni particles

#### Continuous operation mode

**Figure 3:**
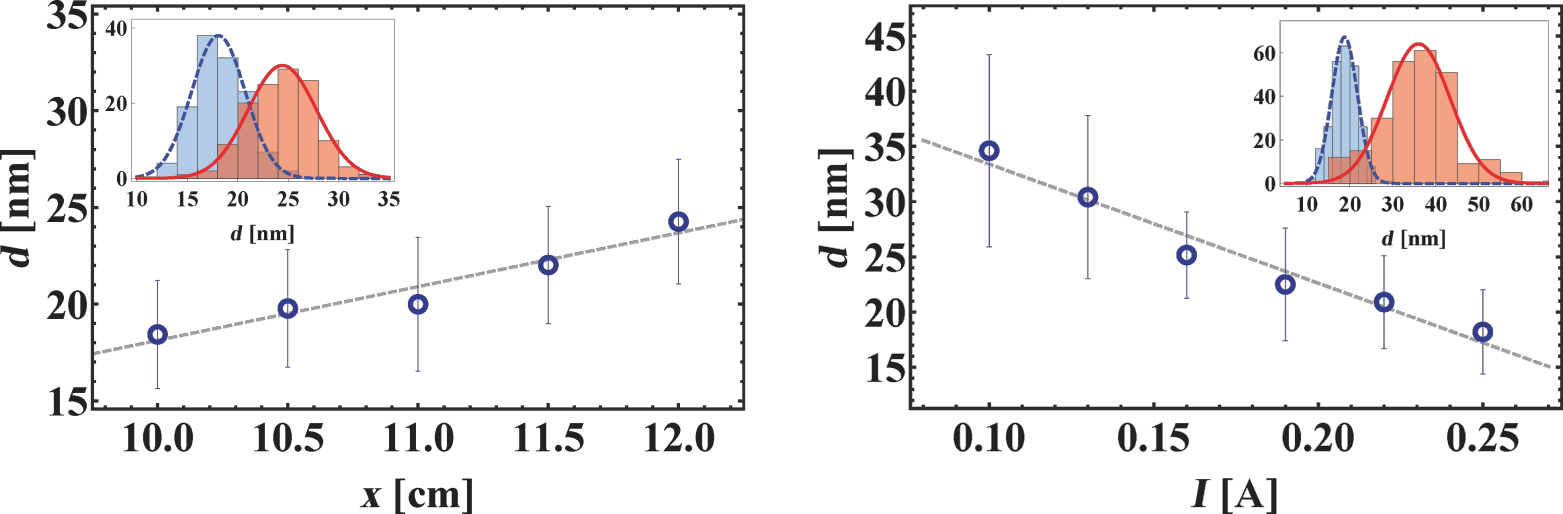
Elemental Ni NP diameter dependency on a) aggregation length *L*1 and b) electrical current of the sputter gun SG_1_. Several hundred particles imaged with SEM have been analyzed by hand to gain satisfactory statistics. Interestingly, particle distributions are close to Gaussian and show little resemblance with log-normal size distributions that have been predicted theoretically.

Elemental magnetic Ni NPs have been generated for various values of pressure and current as well as growth distances by solely using SG_1_ in continuous mode. As seen in Figure 3a, when varying the aggregation length, a clear increase of the particle diameter was found with sizes evolving roughly from 18 to 24 nm at constant pressure. Surprisingly, we observed that crossing a minimum value of *x*, the particle flow ceased abruptly. This has also been reported in earlier studies [13], but the origin of this effect remains unclear to date. In addition to the mean diameter, the particle size distribution has been analyzed. It was found to be close to Gaussian and only slightly skewed, which stands in contrast to results gained with other inert gas condensation techniques like thermal evaporation, for which log-normal size distributions are usually reported [7]. The influence of the gun current on the particle diameter at fixed condensation pressure and aggregation length is presented in Figure 3b. In contrast to previous studies on Ni NPs [13], the latter is found to decrease monotonously with increasing gun current, with maximum average sizes reached in the present study around 35 nm, while raising the current up to 0.25 A results in particles as small as 18 nm. Finally, EDX measurements hint at a small amount of Fe impurities, which could be traced back to undesirable sputtering of the target holder at elevated pressures in chamber A.

#### Target morphology and intermittent mode

**Figure 4:**
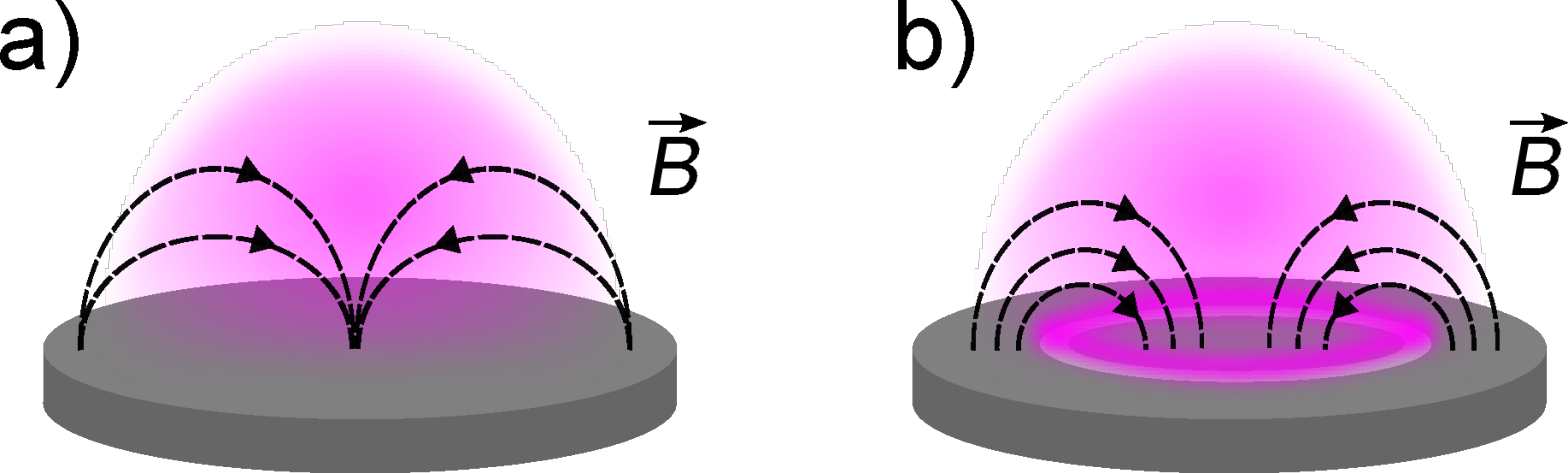
Erosion of the target is found to affect particle sizes and structure. Schematic of the sputtering target a) fresh planar target, b) eroded target eventually generating an increase in plasma density, sputter yield and temperature in the vicinity of the racetrack.

**Figure 5:**
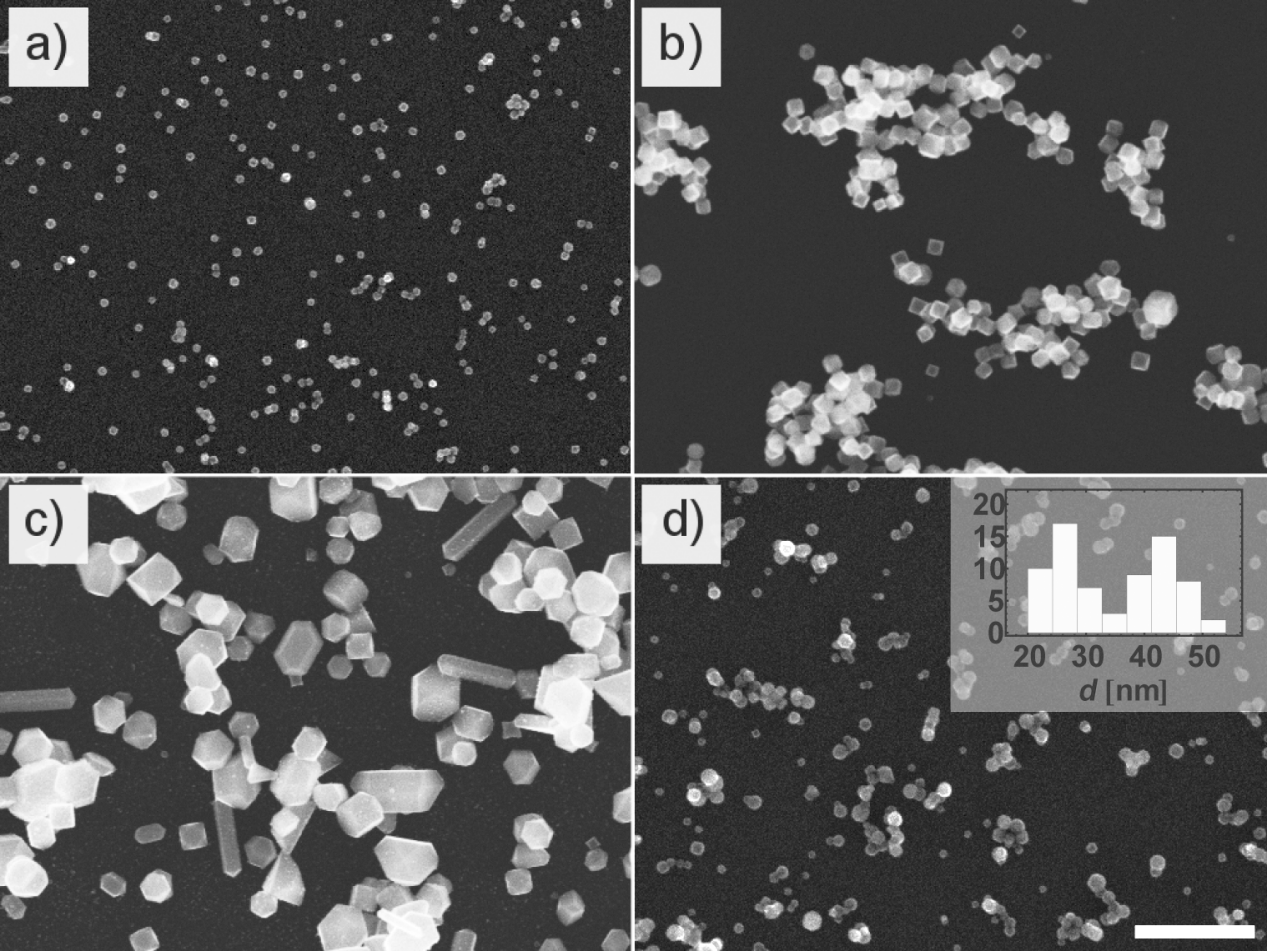
SEM pictures of particles generated with our plasma gas condensation setup. a) Polycrystalline Ni NPs (continuous mode), b) single crystalline Ni NPs produced with strongly eroded target (continuous mode), c) Large sub-micron particles synthesized with strongly eroded target (intermittent mode), d) Ni@Cu CS particles produced by simultaneously using SG_1_ and SG_2_ (continuous mode). All pictures have been taken at the same magnification. The length of the scale bar is 500 nm. (lower right).

Inhomogeneous depletion of the sputtered material in magnetron sputtering sources is known to result in pronounced circular trenches in the target (racetracks), as depicted in Figure 4b. In the present study, this undesirable side-effect was found to have a dramatic influence on the size as well as the morphology of the particles. Indeed, as shown in Figure 5, NPs produced with a strongly used target exhibit typical radii increased by almost an order of magnitude with respect to particles obtained with a planar target at moderate powers (*P* < 60 W).

While the latter typically possess a polycrystalline structure (as will be shown in a later part of this work), larger particles are mostly single crystalline with truncated cubic and cuboctahedral crystal shapes reminiscent of Wulff-construction structures (Figure 5b). When operating the magnetron gun in a high-power (*P* > 400 W) intermittent mode [18] with low-frequency pulses and pauses of the order of 10^−1^ Hz, the particle morphology was found to change dramatically and resulted in a population composed of various sub-micron crystals like rods, triangular plates, pyramids and cubes. While this reminds of earlier studies reporting accurate morphology tuning of Cu crystals by pre-seeded sputtering [18], we stress that present results turned out to be intrinsically linked to the target morphology. Using a planar target in combination with pulsed sputtering did not result in the structures described above. On the contrary, a population of very small particles with radii <10 nm was gained, which again hints at a strong correlation between the target morphology and the growth mechanism of the NP.

### Bimetallic Ni@Cu particles

In order to ensure a good reproducibility and to avoid eventual shortcomings induced by a missing knowledge of the exact target morphology, all bimetallic particles generated in the following study have been produced using planar, non-eroded targets with the magnetron gun operated in continuous mode. Figure 5 shows SEM pictures of a sample generated by using solely SG_1_ in direct comparison with NPs obtained by operating SG_1_ and SG_2_ simultaneously (see Table 1 for operation parameters).

**Table 1:** Optimized operating conditions and synthesis parameters used for the production of CS-NPs described in the present study.

*P*_A_ [mbar]	*P*_B_ [mbar]	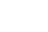 [mol/s]	*I*_SG1_ [mA]	*I*_SG2_ [mA]	*U*_SG1_ [V]	*U*_SG2_ [V]

1.52	0.34	1.3·10^−5^	150	90	368	270

**Figure 6:**
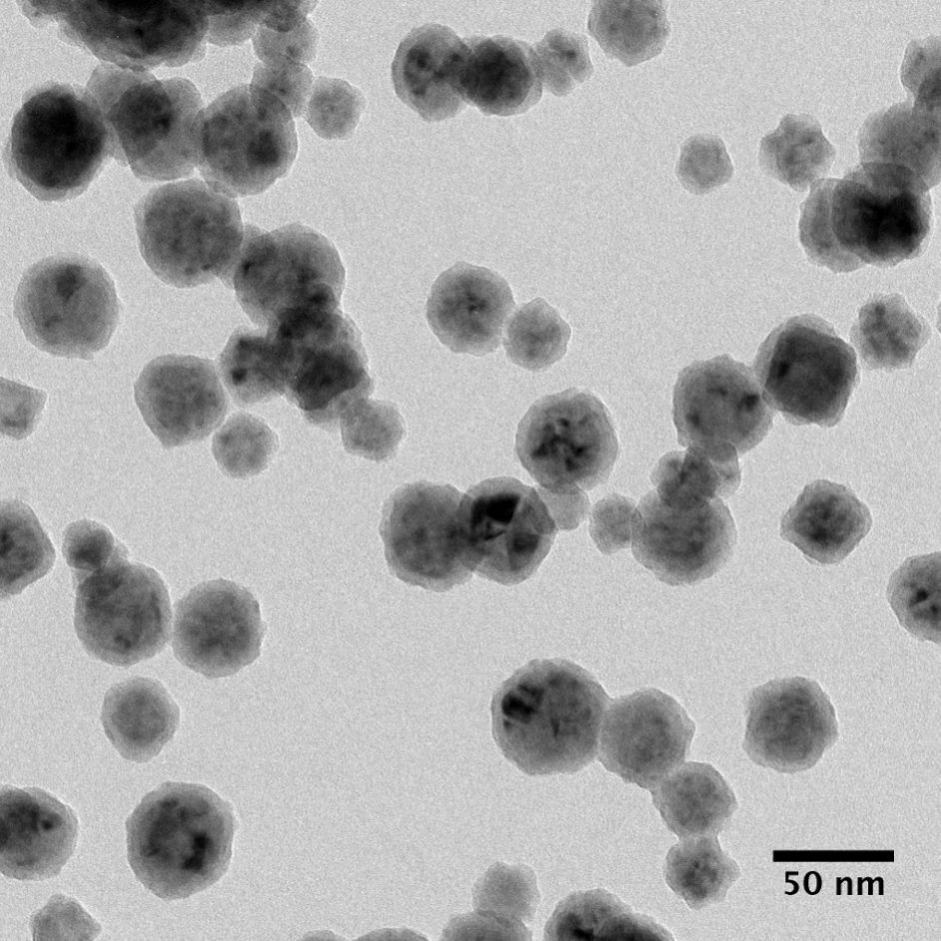
CS-NP deposited on a TEM grid coated with lacey carbon. The same bimodal size distribution as observed in SEM is retrieved.

**Figure 7:**
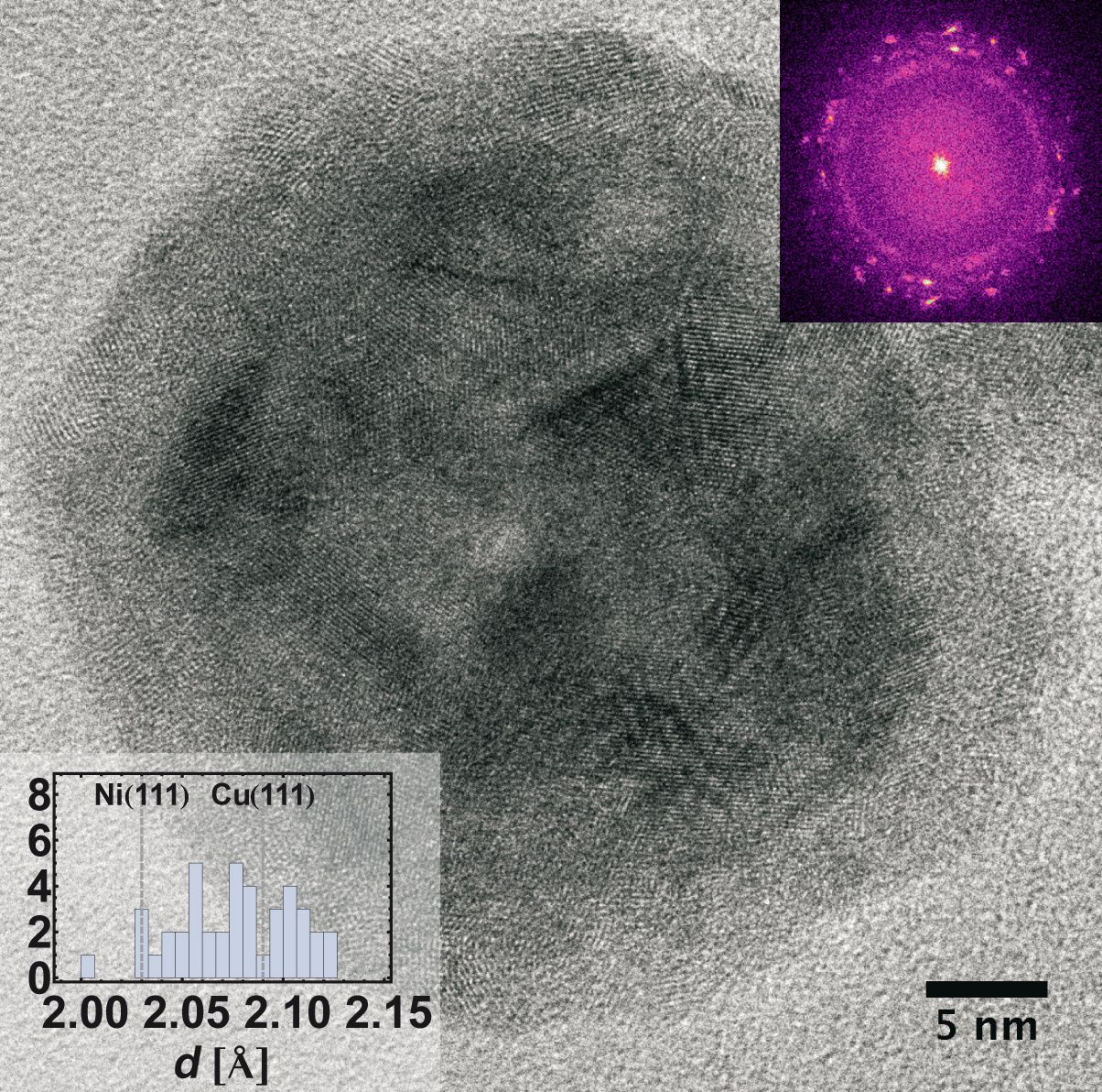
Single CS-NPs exhibit a polycrystalline core as confirmed by FFT analysis (inset upper right) surrounded by an oxidized layer of several nanometers thickness. All particles investigated with HRTEM are found consistent with Cu and Ni fcc phases with a slight systematic shift towards higher values, when compared to bulk plane separations (inset lower left for Cu(111) and Ni(111)).

While in Figure 5a, the NPs exhibit a well defined narrow Gaussian size distribution, analysis of the sizes in Figure 5d on the contrary results in a bimodal distribution. It is also noteworthy that elemental Ni particles possess a rougher surface while all particles with *d* ≥ 30 nm exhibit a more regular and rather smooth contour in the SEM micrographs. In order to assess the relative concentration of the components, SEM-EDX spectra (not shown herein) over areas of approximately 100 μm^2^ have been recorded, which yielded values between Cu_67_Ni_33_ and Cu_49_Ni_51_, depending on the position on the sample. TEM inspection of the particles yields the same binodal size distribution as previously observed with SEM, which becomes apparent in Figure 6. TEM bright field images show that all analyzed NPs present a darker core and brighter halo surrounding the latter. This halo is found to be more pronounced for larger particles and has a typical thickness of 4–5 nm (Figure 7).

To gain quantitative information about the atomic structure of the nanoparticles, lattice plane separations in individual particles were assessed by analysing the FFT of HRTEM micrographs. In a first step, only the core of individual particles was taken into consideration. It was found to be polycrystalline and yielded results in agreement with Cu and Ni fcc phases (JCPDS: 04-0836 *a* = 0.3615 nm and JCPDS: 04-0850 *a* = 0.3523 nm). Although a systematic spread of lattice spacings beyond bulk values was observed, it remained within the estimated uncertainty of the TEM (1.5%). A closer look at the brighter regions surrounding the particles shows that these are composed of small crystallites with typical sizes of a few nm (Figure 7). A FFT of the grains yields lattice spacings that were attributed to Cu_2_O (JCPDS: 05-0667 *a* = 0.4252 nm), although the analysis is seriously impeded by pronounced distortions, small grain sizes and boundaries as well as surface effects.

**Figure 8:**
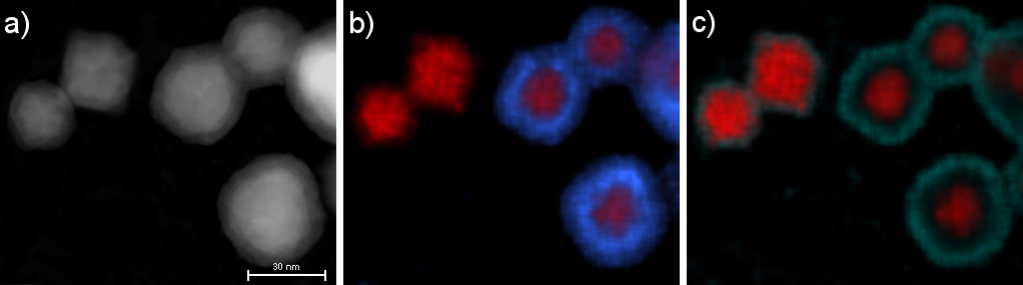
Quantitative EDX mapping revealing the underlying CS-structure: a) HAADF-STEM micrograph, b) Cu (blue) and Ni (red) distribution, c) O (green) and Ni (red) distribution. The length of the scale bar is 30 nm.

Conclusive evidence for the successful synthesis of CS structures is ultimatively provided by EDX mapping as shown in Figure 8. While smaller particles almost exclusively contain Ni, larger NPs present a typical concentric pattern, with a Cu rich shell of approximately 10 nm thickness. Closer analysis shows that this shell consists of an oxygen-rich outer part of several nanometers thickness, which is in agreement with previous HRTEM bright field image results. Finally, the long-term stability of the samples has been analyzed. CS-NPs have therefore been stored for 12 months under ambient air conditions. Subsequent HRTEM and EDX spectra analysis demonstrated the stability of the heterostructured particles, which remained in a CS configuration and showed no indication for enhanced oxidation.

## Discussion

### Elemental particles

Several studies have put under scrutiny the growth mechanisms of elemental NP in plasma gas condensation sources [13,16,19]. Unfortunately, the analysis is often hindered by the lack of information concerning relevant synthesis parameters that are difficult to assess experimentally and can only, if at all, be inferred indirectly. Results found in literature therefore remain contradictory and lack comparability. While some authors found increasing nanoparticle sizes with increasing electrical current of the magnetron gun [13], the results gained with our setup show the opposite trend. Similar findings have been reported recently for the synthesis of niobium NPs [19], in which the temperature profile inside the condensation chamber was taken into account. Local heating of the inert gas through thermalization of the sputtered atoms was argued to be responsible for a nucleation delay, increasing the gun power thereby resulted in a decrease of cluster size. Still, it remains unclear to what extent this explanation is applicable to our setup. Indeed, as described in a previous section, the overall rise of pressure in the condensation chamber measured in the present experiments is small, even at high powers. If inert gas heating through metal vapor thermalization is responsible for this effect, it must be restricted to a region close to the target in order to leave the overall gas temperature nearly constant. Then, its pronounced impact on particle size remains puzzling. Other mechanisms giving rise to a decrease of particle size with increasing gun current are also conceivable. It is known for example from wet-chemistry synthesis of NPs that the degree of supersaturation can be used to tune particle sizes and size distribution. At high supersaturation ratios, fast nucleation rates are attained and the ensuing rapid consumption of the elemental species (nucleation burst) results in a high number of seeds and a fast drop below the nucleation barrier, thus a large number of small particles with narrow size distribution. On the other hand, low supersaturation manifests in a low nucleation rate, larger particles and broader distributions. In this scenario, particle growth would be limited by the available local amount of metal vapor in the particle coordinate system. Unfortunately, it is unknown to date, whether NP production in a cluster source has to be considered a highly localized phenomenon, and additional assumptions concerning the homogeneity of the metal vapor distribution are often plugged ad-hoc into existing growth models. An additional problem that comes into play and has already been discussed in literature [16,19] is the extent of metal vapor loss at the chamber walls. Indeed, although thermalization is believed to be carried out rapidly with regard to typical chamber dimensions, diffusion of metal vapor in the background gas can take place on much shorter time scales than convective transport in the stream. Assuming sticking coefficients close to one, one has to consider the vessel walls as a sink for metal vapor atoms with considerable impact on the resulting steady state vapor density, as will be argued below. Again, a quantitative description of this effect has not yet been achieved.

The dependency of nanoparticle size on increasing growth distance has generated, at least qualitatively, less disagreement. All studies known to us report an increasing particle size with increasing growth distance, in accord with our findings. Nevertheless, robust quantitative analysis of the phenomenon remains difficult. Indeed, all studies have at some point to commit to a specific growth mechanism. Hihara et al. [13] compared growth through collisions via a Smoluchowski equation with addatom growth in Ni particle synthesis and found the latter to be better suited for description of their data. Quesnel et al. [16] also sticked to addatom growth to determine Cu cluster size distributions. While yielding good results for clusters with sizes of several nm, their model failed when applied to NPs with greater average diameters. NPs generated in the present experiments are roughly one order of magnitude larger than those described in the studies mentioned above. This might be traced back to significantly lower particle velocities in the growth region, when comparing to available data. Unfortunately, this severely impedes the description of the NP growth. As it becomes clear from the present TEM results, Ni particles shown herein are highly polycrystalline, suggesting that, at least in later growth stages, collisions and sintering of smaller clusters play a significant role. On the other hand, early growth stages will eventually rely on nuclei growth through deposition of single atoms. This gives rise to additional parameters (like the crossover time *τ*_c_ from single atom deposition to coagulation driven growth) that would be needed for modeling but cannot be assessed with the present setup. This impedes any reasonable analytical description of growth processes in the condensation chamber.

Our results also clearly highlight the influence of target morphology on particle growth. As was shown in earlier studies, shaping the plasma above the target by manipulation of the magnetic field can have a strong effect on particle structure and phase [30]. Here, a similar trend is observed, although additional mechanisms might play a role. Indeed, the appearance of a profound racetrack might not only change the magnetic field configuration and thereby induce an increase in plasma density near the target (Figure 4). The creation of a curved surface can also dramatically enhance the sputter yield, which is known to be severely affected by the incidence angle of the impinging Ar ions, an effect which has been shown to influence the size of NPs generated in plasma condensation sources [31]. Further experimental investigation is needed to clarify the exact correlation of target shape and produced NPs.

### Bimetallic nanoparticles

Despite all the shortcomings described before, metallic shell addatom growth onto particles traveling through the gas phase seems to be, in principle, amenable to analytical modeling. As long as nucleation of the shell species is avoided, expressions for the size evolution of CS-NPs in the coating chamber can be derived by using classical kinetic gas theory, as the particle size is well below the typical mean free path of the background gas atoms. Assuming the traveling velocity of the NPs to be identical to the gas stream velocity, the growth of a shell onto an existing core can be described by [32]:

[4]
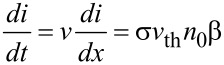


Here, *i* represents the number of shell atoms, *x* the traveling distance through the coating chamber B, *v*_th_ = 
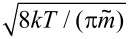
 the thermal velocity in the gas phase with 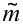 the reduced mass of the collision partners, *σ* = *π* (*r*_p_ + *r*_Cu_)^2^ the interaction cross section of a particle with radius *r*_p_ with a Cu atom with radius *r*_Cu_ and finally *n*_0_ the density of thermalized Cu atoms in the gas phase. A possible re-evaporation of deposited shell atoms is taken into account by the factor *β* and plays a major role in initial stages of cluster growth, as the large heat of condensation of metals can significantly reduce the sticking rate of impinging atoms on small clusters [13,32]. Nevertheless, in the present study, due to the rather large size of the Ni core, evaporation rates will be assumed close to zero, setting *β* equal to one. Additionally, the following approximations are used: 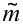 ≈ *m*_Cu_ and *r*_p_ + *r*_Cu_ ≈ *r*_p_. This yields

[5]
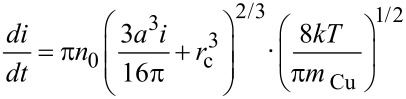


This differential equation is easily solved by using appropriate boundary conditions. Unfortunately, the vapor density is a crucial parameter that can only be determined indirectly. It has recently been proposed to use a simple balance equation for the calculation of *n*_0_ [16]. Equating the incoming metal atom flux from the sputter gun with the outflowing metal vapor yields


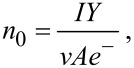


where *I* denotes the electrical current of the sputter gun, *Y* the sputter yield of the Cu target, *v* the mean convective velocity of the gas, *A* the average area of the vessel B and *e*^−^ the electron charge. Applying this to our setup (*Y* = 1.2, *I* = 0.09 A, *v* = 0.028 m·s^−1^, *A* = 3.1·10^−2^ m^2^) yields *n*_0_ ≈ 7.5·10^20^ m^−3^. On the other hand, the vapor density can also be deduced from the effective shell thickness by using the above equation (*a* = 0.362·10^−9^ m, *v* = 0.028 m·s^−1^, *r*_c_ = 12.5·10^−9^ m, *x* = 0.2 m), which results in: *n*_0_ ≈ 1.5·10^18^ m^−3^. This large discrepancy clearly illustrates the shortcomings of modeling NP growth. Lower metal vapor densities allow two interpretations. Either our setup faces a large loss of metal vapor at the chamber walls, an assumption which is supported by calculations of Cu diffusivity in buffer Ar by using the Chapman–Enskog theory [33], or the sputter rate is incorrectly determined by neglecting collisional processes of Ar-ions on their way to the target. This shows that, in order to gain a better quantitative understanding of heterostructured NP growth, direct assessment of metal vapor densities is a crucial requirement. Experiments aiming at a determination of *n*_0_ are currently underway.

With respect to the structure of the CS particles, several things are noteworthy. No elemental Cu particles were found in the present experiments. This was realized by carefully working at sufficiently low pressures and demonstrates the possibility of a complete suppression of homogeneous nucleation of the shell species in the gas phase. Unfortunately, the presence of shadowed or blind zones in the vessel, where Ni core particles can pass without encountering sufficient Cu atoms lead to a significant amount of uncoated NPs. This can in principle be avoided by an improved design of the coating chamber.

Finally, as discussed above, oxidation of the NPs surface was observed, but restricted to a thin layer of few nanometers thickness, for CS as well as for elemental particles. This can be either attributed to the presence of residual oxygen in the chamber, which would require the usage of even higher gas purities in order to avoid contamination, or the transport of the particles through air for further analysis. Future studies will aim at circumventing this problem by using a noble metal as the shell material.

## Conclusion

We set up a plasma gas-condensation apparatus for the synthesis of nanoparticles and demonstrated the successful production of highly tunable elemental and CS-structured magnetic nanoparticles. In contrast to earlier studies, in which a shell thickness of only several monolayers was reached [10], the present experiments demonstrate the capacity of plasma gas condensation to synthesize CS-NPs with much larger shell dimensions. Deposition of a 10 nm layer of Cu atoms on Ni cores was explicitly shown by using HRTEM and EDX. Repetition of the measurements showed that particle structures remained stable under ambient air conditions for a period of more than 12 months. A description of the shell growth by using simple kinetic gas theory arguments was undertaken and demonstrated the importance of an exact knowledge of metal vapor densities in the chamber to understand addatom deposition. By using the particles as local probes, the gas phase density, *n*_0_, was assessed and shown to differ dramatically from results gained from simple flux calculations. This might be attributed to a) oversimplifying assumptions concerning the sputter rates or b) significant losses of metal vapor at the chamber walls. Finally, we emphasize that, although the present study was restricted to Cu/Ni as a material system, the growth of fine tuned shells by using gas-phase magnetron deposition is generalizable to any sputterable material, thus paving the way for synthesis of novel multifunctional NPs.
